# Patient Attitudes Toward Telepsychiatry During the COVID-19 Pandemic: A Nationwide, Multisite Survey

**DOI:** 10.2196/24761

**Published:** 2020-12-22

**Authors:** Daniel Guinart, Patricia Marcy, Marta Hauser, Michael Dwyer, John M Kane

**Affiliations:** 1 Department of Psychiatry The Zucker Hillside Hospital Northwell Health New York, NY United States; 2 Center for Psychiatric Neuroscience Feinstein Institutes for Medical Research Manhasset, NY United States; 3 The Donald and Barbara Zucker School of Medicine at Hofstra/Northwell Manhasset, NY United States; 4 Vanguard Research Group Glen Oaks, NY United States; 5 Ambulatory Care Division The Zucker Hillside Hospital Northwell Health New York, NY United States

**Keywords:** telehealth, telepsychiatry, telemedicine, attitude, patients, survey, COVID-19, mental health

## Abstract

**Background:**

The COVID-19 pandemic and its associated movement restrictions forced a rapid and massive transition to telepsychiatry to successfully maintain care continuity.

**Objective:**

The aim of this study is to examine a large number of patients’ experiences of, use of, and attitudes toward telepsychiatry.

**Methods:**

An anonymous 11-question survey was delivered electronically to 14,000 patients receiving telepsychiatry care at 18 participating centers across 11 US states between the months of April and June 2020, including questions about their age and length of service use, as well as experience and satisfaction with telepsychiatry on a 5-point Likert scale. Descriptive statistics were used to analyze and report data.

**Results:**

In total, 3070 patients with different age ranges participated. The overall experience using telepsychiatry was either excellent or good for 1189 (82.2%) participants using video and 2312 (81.5%) using telephone. In addition, 1922 (63.6%) patients either agreed or strongly agreed that remote treatment sessions (telephone or video) have been just as helpful as in-person treatment. Lack of commute (n=1406, 46.1%) and flexible scheduling/rescheduling (n=1389, 45.5%) were frequently reported advantages of telepsychiatry, whereas missing the clinic/hospital (n=936, 30.7%) and not feeling as connected to their doctor/nurse/therapist (n=752, 24.6%) were the most frequently reported challenges. After the current pandemic resolves, 1937 (64.2%) respondents either agreed or strongly agreed that they would consider using remote treatment sessions in the future.

**Conclusions:**

Telepsychiatry is very well perceived among a large sample of patients. After the current pandemic resolves, some patients may benefit from continued telepsychiatry, but longitudinal studies are needed to assess impact on clinical outcomes and determine whether patients’ perceptions change over time.

## Introduction

The outcomes and cost-effectiveness of telepsychiatry are overall comparable to in-person care across multiple treatment modalities, disorders, and patient groups [1-11]. However, widespread implementation of telepsychiatry has been challenging [12-14], partially due to mental health care professionals’ concerns about patients’ ability to use conferencing devices, lack of sense of closeness/connection, technical problems, and reimbursement and privacy concerns [15,16]. However, barriers related to patient preference are also possible, and the patient perspective is crucial to further characterize implementation challenges. Previous studies showed positive patient satisfaction [17-19] but potential limitations including relatively small sample sizes and/or selection biases in the context of pilot programs or specific services may have limited their generalizability.

Due to the COVID-19 crisis, our health care system and others around the world rapidly transitioned all or almost all in-person visits to remote assessments [20], in an unprecedented context of mental health care professional stress and increased need for mental health services [21,22]. This revolution in telepsychiatry provided a unique opportunity to assess how patients that may not have initially opted for telepsychiatry feel about it. Hence, the aim of this study was to qualitatively assess opinions and attitudes about telepsychiatry of a large sample of patients.

## Methods

In collaboration with the Vanguard Research Group (VRG), a research consortium specializing in behavioral health, an anonymous survey was distributed to patients using telepsychiatry in 18 hospitals and community centers located in rural, suburban, small urban, and large urban areas in 11 different states across the United States (Connecticut, Florida, Maine, Michigan, New Hampshire, New York, Oregon, Rhode Island, South Carolina, Texas, and Utah). Surveys were distributed through email and/or embedded into the video platform scheduling invitations between the months of April and June 2020, and could be completed electronically, with computers, tablets, or smartphones. Study procedures were deemed exempt by the local Institutional Review Board (IRB#20-0397). Further details can be found on the Checklist for Reporting Results of Internet E-Surveys (CHERRIES) [23] listed in Multimedia Appendix 1.

The survey included 11 questions about telepsychiatry use and satisfaction using a 5-point Likert scale, as well as inquiries about both potential challenges and positive experiences (see survey in Multimedia Appendix 2). Descriptive statistics were used to report qualitative survey results. Chi-square tests were used to compare categorical variables. First, omnibus comparisons were conducted by age range and length of care at the same institution. If statistically significant differences were detected, we then tested the individual interactions of interest post hoc. All analyses were conducted using JMP (Version 13, SAS Institute Inc).

## Results

The survey was distributed to approximately 14,000 patients, of which 3070 (22%) completed it. In total, 18 surveys were excluded due to the subject disclosing not having used telemedicine. Hence, 3052 surveys were included in the analysis. Patient characteristics are listed in Table 1.

**Table 1:** Characteristics of the patients included in the study.

Characteristics		Patients, n (%)
**Age range, years (N=3040)**		
	<25	304 (10.0)
	25-34	494 (16.3)
	35-44	576 (18.9)
	45-54	680 (22.4)
	55-64	721 (23.7)
	65-74	232 (7.6)
	>74	33 (1.1)
**Duration of care, years (N=2994)**		
	<1	793 (26.5)
	1-5	1335 (44.6)
	5-10	493 (16.4)
	>10	373 (12.5)

Briefly, 55% of the sample (n=1666) were aged >45 years and the majority of participants (n=2128, 71.1%) had been under care at the institution where the survey took place for ≤5 years (Table 1). Respondents were mostly using telephone (n=1924, 63.7%), followed by video (n=708, 23.4%), and a combination of telephone and video (n=390, 12.9%). When asked about their preferred method of receiving care, respondents preferred the telephone over video (n=1908, 64.1% versus n=1066, 35.9%).

**Figure 1:**
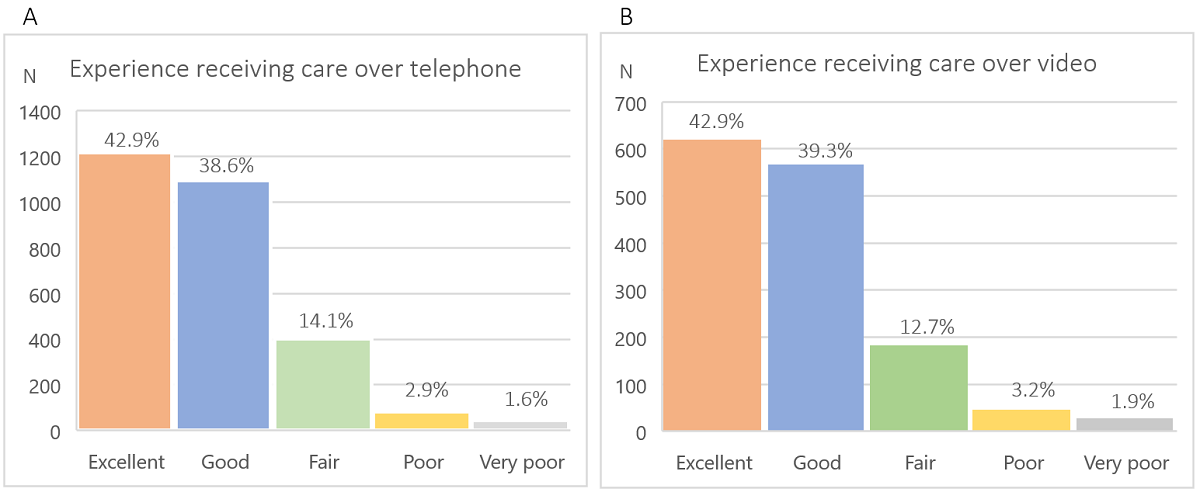
(A) Patients' experience of receiving mental health care via telephone, rated on a 5-point Likert scale ranging from 0=very poor to 5=excellent. (B) Patients' experience of receiving mental health care via video, rated on a 5-point Likert scale ranging from 0=very poor to 5=excellent.

The overall experience was either good or excellent for 2312 (81.5%) of respondents when asked about telephone only and for 1189 (82.2%) when asked about video (Figure 1). Only 127 (4.5%) and 74 (5.1%) respondents rated their experience as “poor” or “very poor” for telephone and video, respectively (Figure 1).

We detected differences in the overall experience by age range in the case of telephone, χ²_24_ (n=2831)=46.3, *P*=.004; a lower proportion of patients aged 55-64 years declared their experience as excellent compared to other age groups (n=257, 38.2% versus n=960, 44.3%), χ²_4_ (n=2840)=12.8, *P*=.01. In addition, a higher proportion of patients aged 45-54 years rated their experience as poor compared to other age groups (n=27, 4.2% versus n=55, 2.5%), χ²_4_ (n=2840)=10.5, *P*=.03.

**Figure 2:**
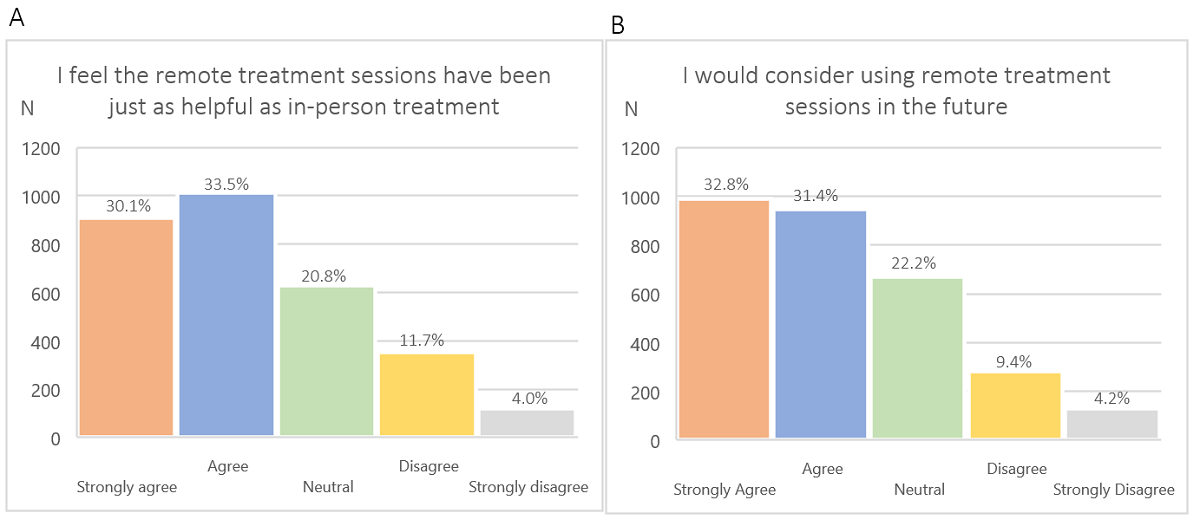
(A) Patients' degree of agreement with the statement "I feel the remote treatment sessions have been just as helpful as in-person treatment,” rated on a 5-point Likert scale ranging from "strongly disagree" to "strongly agree." (B) Patients' degree of agreement with the statement "I would consider using remote treatment sessions in the future," rated on a 5-point Likert scale ranging from "strongly disagree" to "strongly agree.".

Further, 1922 (63.6%) patients either agreed or strongly agreed with the statement that remote treatment sessions (telephone or video) have been just as helpful as in-person treatment, whereas 1937 (64.2%) of respondents either agreed or strongly agreed with the statement that they would consider using remote treatment sessions in the future (Figure 2). Patients using video were more likely to strongly agree with that statement than those using telephone (n= 570, 38.9% versus n= 273, 29.9%), χ²_4_ (n=2605)=29.6, *P*<.001.

Patients endorsed the lack of commute (n=1406, 46.1%), flexible scheduling/rescheduling (n=1389, 45.5%), reduced likelihood of missing appointments (n=1064, 39.9%), and feeling more confidence/comfort than in person (n=601, 19.7%) as positive elements/advantages of telepsychiatry (Table 2), which did not vary by age (χ²_18_ [n=4447]=15.4, *P*=.64) or length of time under care (χ²_9_ [n=4413]=10.7, *P*=.30). Some of the challenges that patients endorsed were related to missing the clinic/hospital (n=936, 30.7%) and not feeling as connected to their doctor/nurse/therapist (n=752, 24.6%), among others (Table 2). Patients under care for less than one year endorsed missing the clinic and feeling connected to it less frequently than other groups (n=195, 21.6% versus n=741, 28%), χ²_6_ (n=3550)=21.5, *P*=.002.

**Table 2:** Patient-reported advantages and challenges related to the use of telepsychiatry (N=3052).

Advantages and challenges		Participants, n (%)^a^
**Positive elements of telepsychiatry**		
	I like not having to commute to the clinic	1406 (46.1)
	Flexible scheduling/rescheduling	1389 (45.5)
	I am less likely to miss appointments	1064 (34.9)
	I felt more confident/comfortable than in person	601 (19.7)
**Difficulties and challenges of telepsychiatry**		
	I miss visiting the clinic/hospital and feeling connected to it	936 (30.7)
	I do not feel as connected to my doctor/nurse/therapist	752 (24.6)
	I am concerned that my doctor/nurse/therapist might miss something because they do not see me in person (eg, a side effect of the medicine)	593 (19.4)
	I do not feel as comfortable talking about my problems as I do in person	471 (15.4)
	I have had technical problems establishing/maintaining the connection	375 (12.3)
	I am concerned about confidentiality/privacy	273 (8.9)
	I do not feel that my doctor/nurse/therapist is as engaged in the conversation	150 (4.9)

^a^Percentages represent the proportion of responders who endorsed a given option and are calculated in relation to the total number of respondents, since more than one positive element and/or challenge or difficulty could be selected. Responses are listed in order of most frequently endorsed items.

In the free-text comment section, patients generally found telepsychiatry to be safe and convenient, and expressed their gratitude to mental health care professionals for providing uninterrupted care during a very challenging time. Many suggested remote assessments should be maintained, mentioning that they feel more comfortable at home, can express themselves more freely, save transportation time and costs, and/or request less time off work. Others expressed feeling disengaged, feeling frustrated with technical difficulties and having a lack of resources to address them (eg, not owning a laptop or smartphone), difficulty finding a quiet setting (eg, children interrupting, shared housing), getting tests done or filling out forms.

## Discussion

### Principal Findings

In this study, we report highly favorable attitudes toward telepsychiatry in its diverse forms, across a large sample of patients across the United States. To our knowledge, this is the largest evaluation of patient attitudes toward telepsychiatry to date, by at least an order of magnitude, which is timely in the context of the current COVID-19 pandemic and the widespread stay-at-home and travel restriction orders, the duration of which is unclear.

Our results are aligned with other surveys very recently validated based on quality of care domains [24], showing high levels of satisfaction with telepsychiatry services. Other recent studies in older [25,26] and younger [27] adults showed similar results, all in smaller samples. Further, most of our respondents would like to continue using telepsychiatry. This finding is highly relevant given the diversity and size of our sample, drawn from a large network of community, real-world, and academic mental health centers, and should encourage allowing telepsychiatry to continue for some patient populations after the current pandemic is resolved. However, some respondents expressed a desire to resume usual in-person care as soon as possible and/or lean toward hybrid models. The option of telepsychiatry should remain tailored to individual patient needs and be the result of shared decision making.

Interestingly, subjects were more likely to strongly agree to consider using telepsychiatry in the future when using video. Concerns raised about lack of closeness and fear of a reduction in the doctor’s ability to detect subtle signs of body language, nonverbal cues, and/or physical signs of disease could be some of the reasons behind this preference [16]. Whereas the widespread use of the telephone may be the result of an abrupt transition related to COVID-19, access to technology may have been a potential barrier to the implementation of telepsychiatry that will need to be considered. Videoconferencing should be preferred over telephone whenever possible, particularly given the currently available technology, which allows for encrypted private communications [15]. Further, patients with sensory and/or cognitive limitations such as mutism, hearing difficulty, or visual or cognitive impairment would potentially require deployment of additional technologies and/or human resources.

### Limitations

This study has some limitations. First, this study was conducted during the COVID-19 pandemic and associated movement restrictions, which may have made hospital/doctor visits less appealing, adding safety as a confounder, possibly overestimating real user satisfaction. Second, our survey was short, the completion rate was relatively low, and our sample was not random, so selection, nonresponse, and response biases are possible [28]. Third, the influence of additional sociodemographic factors as well as symptom severity and/or previous telepsychiatry experience could not be ascertained. Longitudinal studies will be needed to assess impact on clinical outcomes and determine whether patients’ perceptions change over time.

Mental health professionals were already implementing digital technologies and advocating for more widespread use of telehealth [29], and the current scenario has accelerated its use. Thus, even after the COVID-19 pandemic ends, telepsychiatry is here to stay. However, patient concerns need to be heard and addressed, and positive experiences need to be acknowledged and echoed.

### Conclusion

Patients had a generally positive attitude toward telepsychiatry and many would like to continue using it after the COVID-19 restrictions recede. Longitudinal studies are needed to assess whether patient perceptions change over time. However, some patients may benefit from continuous use of telepsychiatry. Results of this study should help shape policies regarding its use.

## Appendix

Multimedia Appendix 1 Checklist for Reporting Results of Internet E-Surveys (CHERRIES).

Multimedia Appendix 2 Telepsychiatry Patient Satisfaction Survey.

## Abbreviations

CHERRIES: Checklist for Reporting Results of Internet E-Surveys
