# Supplementary material for: Patient Attitudes Toward Telepsychiatry During the COVID-19 Pandemic: A Nationwide, Multisite Survey
Source: JMIR Ment Health. 2020 Dec 22;7(12):e24761. doi: 10.2196/24761 (PMC7758084; doi:10.2196/24761)
Supplement: Multimedia Appendix 1 [file mental_v7i12e24761_app1.pdf]

Checklist for Reporting Results of Internet E-Surveys (CHERRIES)		
Item Category	Checklist Item	Explanation
Design	Survey design	An anonymous survey was distributed electronically among all patients receiving telepsychiatry (convenience sample)
IRB approval and informed consent process	IRB approval	Study procedures were deemed exempt by the local Institutional Review Board (IRB#20-0397)
	Informed consent	Consent language was embedded in the survey as shown in Supplement 2
	Data protection	No protected health information (PHI) or personal information was collected, and age was only collected in age ranges. Data was not shared or transmitted to any third entity
Development and pretesting	Development and pretesting	An electronic questionnaire was developed by our team based on literature and clinician feedback. Data collection and technical functionality were tested in-house before field deployment
Recruitment process and description of the sample having access to the questionnaire	Open survey versus closed survey	The survey was open, but links to the survey were distributed only to patients receiving telepsychiatry
	Contact mode	Surveys were distributed through email and/or embedded into the telepsychiatry video platform scheduling invitations
	Advertising the survey	The survey was not advertised
Survey administration	Web/E-mail	The survey is posted on a site. Responses are captured automatically, eliminating risk of data loss or mishap
	Context	The survey was self-standing and there is no influence of other contents on the webpage
	Mandatory/Voluntary	Survey was voluntary
	Incentives	There were no incentives offered
	Time/Date	April - June of 2020

	Randomization of items/questionnaires	Items were not randomized
	Adaptive questioning	Items were not adaptive
	Number of items	11 items
	Number of pages	Items were displayed in one single page
	Completeness check	Items provided a non-response and/or open-ended option, except for 5-point Likert scale options that were enforced
	Review step	Respondents were able to review and change their answers before submission
Response rates	Unique site visitor	Only one administration per subject was allowed
	View rates	The survey is sent individually only to patients using telepsychiatry, not located on a public website
	Participation and completion rates	Since items were enforced, there are no measures of participation and completion rate
Preventing multiple entries from the same individual	Cookies/IP check	Only one administration per subject was allowed, but multiple entries could not be enforced, as we did not collect IP or cookies for privacy reasons
	Log file analysis	Since we collected date and time the test was completed, we analyzed the log file to detect multiple entries
	Registration	N/A
Analysis	Handling of incomplete questionnaires	Incomplete questionnaires were included - each survey item was analyzed individually
	Atypical timestamp	Time needed to complete the test was recorded. No specific timeframe was used as acceptability cutoff point due to the short length of the survey
	Statistical correction	Descriptive statistics were used to report qualitative survey results. Chi-Square tests were used to compare categorical variables
