# Supplementary material for: Patient Attitudes Toward Telepsychiatry During the COVID-19 Pandemic: A Nationwide, Multisite Survey
Source: JMIR Ment Health. 2020 Dec 22;7(12):e24761. doi: 10.2196/24761 (PMC7758084; doi:10.2196/24761)
Supplement: Multimedia Appendix 2 [file mental_v7i12e24761_app2.pdf]

Telepsychiatry Patient Satisfaction Survey

The current crisis has resulted in our using telehealth (video or telephone) to continue our treatment sessions with you. We are interested in learning how you feel about this change in treatment and whether we should consider using this more in the future. This is a research study. Participation in this survey is voluntary. If you would prefer not to participate, simply do not complete the survey. Your responses are anonymous; do not put your name or other identifying information on this survey. We ask that you try to answer all questions. However, if there are any questions that you would prefer to skip, simply leave the answer blank. We thank you for taking the time to complete this survey.

1. Please select your age range

- ○ <25
- ○ 25-34
- ○ 35-44
- ○ 45-54
- ○ 55-64
- ○ 65-74
- ○ >74

2. I have been receiving care in this hospital/center for:

- ○ Less than one year
- ○ One to five years
- ○ Five to ten years
- ○ More than ten years

3. I am currently using the following method to talk to my doctor/nurse/therapist:

- ☐ Telephone (no real time video)
- ☐ Video platform-Zoom
- ☐ Video platform-Amwell
- ☐ Video platform-Other
- ☐ Telephone (no video) sometimes, video other times.

4. Which of the following methods do you prefer/like the most?

- ○ Telephone (no real time video)
- ○ Video

5. How would you describe your patient experience when using telephone only?

- ○ Excellent
- ○ Good
- ○ Fair
- ○ Poor

- Very poor
- N/A - I have not used telephone only

6. How would you describe your patient experience using video?

- Excellent
- Good
- Fair
- Poor
- Very poor
- N/A - I have not used video

7. I feel the remote treatment sessions (telephone or video) have been just as helpful as in person treatment:

- Strongly agree
- Agree
- Neutral
- Disagree
- Strongly disagree

8.What were some of the challenges you experienced? (check all that apply)

- I do not feel as connected to my doctor/nurse/therapist
- I do not feel that my doctor/nurse/therapist is as engaged in the conversation
- I am concerned that my doctor/nurse/therapist might miss something because they do not see me in person (for example, a side effect of the medicine)
- I do not feel as comfortable talking about my problems as I do in person
- I miss visiting the clinic/hospital and feeling connected to it
- I am concerned about confidentiality/privacy
- I have had technical problems establishing/maintaining the connection
- Other

9.What were some of the positive things you experienced? (check all that apply)

- I feel more confident/comfortable than in person
- I like not having to commute to the clinic
- Flexible scheduling/rescheduling
- I am less likely to miss appointments
- Other

10. I would consider using remote treatment sessions (telephone or video) in the future:

- Strongly Agree
- Agree
- Neutral
- Disagree
- Strongly disagree

11.Is there anything else you would like to tell us?

Thank you for completing the survey.
